# Statistical principle-based approach for gene and protein related object recognition

**DOI:** 10.1186/s13321-018-0314-7

**Published:** 2018-12-17

**Authors:** Po-Ting Lai, Ming-Siang Huang, Ting-Hao Yang, Wen-Lian Hsu, Richard Tzong-Han Tsai

**Affiliations:** 1 Department of Computer Science, National Tsing-Hua University, Hsinchu, Taiwan; 2 Intelligent Agent Systems Laboratory, Institute of Information Science, Academia Sinica, Taipei, Taiwan; 3 Bioinformatics Program, Taiwan International Graduate Program, Institute of Information Science, Academia Sinica, Taipei, Taiwan; 4 Institute of Biomedical Informatics, National Yang Ming University, Taipei, Taiwan; 5 Intelligent Information Service Research Laboratory, Department of Computer Science and Information Engineering, National Central University, Taoyuan, Taiwan

**Keywords:** Named entity recognition, Information extraction, Natural language processing, Biomedical text mining, Machine learning, Medical chemical patent

## Abstract

The large number of chemical and pharmaceutical patents has attracted researchers doing biomedical text mining to extract valuable information such as chemicals, genes and gene products. To facilitate gene and gene product annotations in patents, BioCreative V.5 organized a gene- and protein-related object (GPRO) recognition task, in which participants were assigned to identify GPRO mentions and determine whether they could be linked to their unique biological database records. In this paper, we describe the system constructed for this task. Our system is based on two different NER approaches: the statistical-principle-based approach (SPBA) and conditional random fields (CRF). Therefore, we call our system SPBA-CRF. SPBA is an interpretable machine-learning framework for gene mention recognition. The predictions of SPBA are used as features for our CRF-based GPRO recognizer. The recognizer was developed for identifying chemical mentions in patents, and we adapted it for GPRO recognition. In the BioCreative V.5 GPRO recognition task, SPBA-CRF obtained an F-score of 73.73% on the evaluation metric of GPRO type 1 and an F-score of 78.66% on the evaluation metric of combining GPRO types 1 and 2. Our results show that SPBA trained on an external NER dataset can perform reasonably well on the partial match evaluation metric. Furthermore, SPBA can significantly improve performance of the CRF-based recognizer trained on the GPRO dataset.

## Introduction

The large number of chemical and pharmaceutical patents have prompted active research in biological text mining. Named entity recognition (NER) is a fundamental task in biomedical text mining involving extraction of words or phrases that refer to specific entities, such as genes, diseases and chemicals. The BioCreative V.5 gene and gene product (GPRO) recognition task [1] was designed to promote the development and evaluation of information extraction systems for recognition of GPRO mentions in patents.

In the task, given a patent abstract, a text mining system should identify the boundaries of GPRO mentions in the text (the span) and classify the mentions’ types. The eight GPRO mention types (Table 1) were defined according to users’ requirements. Since the fine-grained mention types were too complicated for the development of NER systems, the task was simplified by merging the eight types into two: GPRO Type 1 and GPRO Type 2. Type 1 mentions can be linked to specific biological database records such as SwissProt and EntrezGene IDs, while Type 2 mentions cannot be linked to unique IDs.

**Table 1:** Example of each GPRO mention type

Original mention type	Simplified mention type	Example
Abbreviation	Type 1	*“TNF”* and *“LFA*-*3”*
Full name	Type 1	*“Cholesteryl ester transfer protein”*
Identifier	Type 1	*“EC 3.4.14.5”*
Nested mentions	Type 1	*“neurokinin*-*1 (NK*-*1) receptor”*
Family	Type 2	*“heparan sulfate binding proteins”*
Multiple	Type 2	*“melanocortin 1 and/or 4 receptors”*
No class	Type 2	*“SH2 domain”*
Sequence	Type 2	*“ACACCUGGUGACUAGUGGUGCG”*

The GPRO task is more challenging than other gene mention recognition tasks, like JNLPBA [2] and Biocreative II GM [3], in the following two aspects.

First, the words surrounding a gene mention may or may not be part of the related GPRO mention. For example, given two phrases *“… VLA*-*4 receptors…”* and *“… A2A receptors…,”* the gold-standard GRPO spans would be *“…* ***VLA-4 receptors***_***GPRO_TYPE_1***_ *receptors…”* and *“…* ***A2A receptors***_***GPRO_TYPE_1***_*…,”* instead of *“…* ***VLA-4***_***GPRO_TYPE_1***_*…”* and *“…* ***A2A receptors***_***GPRO_TYPE_1***_*….”* This is because the spans of GPRO mentions are highly related to biological database records. In the above cases, *“A2A”* is a subtype of “adenosine receptor”. Therefore *“A2A receptors”* could be linked to unique UniProt ID:P29274. However, *“VLA*-*4 receptors”* conjugated from several small subunits but served as a specific protein molecule. Therefore, *“VLA*-*4 receptors”* could not be linked to unique UniProt ID.

Second, two GPRO mentions whose names follow similar conventions may still be different mention types. For instance, the GPRO mentions *“IL*-*2”* and *“CD4”* are distinct proteins that can be linked to corresponding unique UniProt IDs, and therefore belong to Type 1. However, *“IL*-*12”* and *“CD3”* are protein families and thus cannot be linked to unique UniProt IDs, making them Type 2 GPRO mentions.

In this study, we have developed a GPRO recognizer which combines two different approaches: the statistical principle-based approach (SPBA) and conditional random fields (CRF). To tackle the difficult challenge of identifying GPRO mention boundaries in the text, we divide the span recognition problem into two subtasks. In the first subtask, we develop a gene mention recognizer which outputs longer and more consistent gene mention spans. The spans of these mentions are not influenced by whether the mentions can be linked to a biological database or not. We use SPBA to solve this subtask. For instance, in the above example, SPBA would predict *“VLA*-*4 receptors”* and *“A2A receptors”* as gene mentions by labeling them as *“…* ***VLA-4***_***GeneSymbol***_ ***receptors***_***ProteinKeyword***_*…”* and *“…* ***A2A***_***GeneSymbol***_ ***receptors***_***ProteinKeyword***_*…”* according to the pattern “[GeneSymbol][ProteinKeyword].” In the second subtask, we use CRF and post-processing rules to adjust gene mention spans to fit the GPRO annotation standards.

For the second challenge, we have tried two different approaches. First, we treat the two mention types as entirely different named entity types, and use CRF to learn and predict them. Second, we use heuristic rules to predict the mention types of GPRO mentions according to whether they could be linked to unique biological database records or not.

In the BioCreative V.5 GPRO task, our best configuration uses SPBA-CRF. It achieves an F-score of 73.73% on GPRO Type 1, which is ranked the 4th place on the task, and an F-score of 78.66% on GPRO Types 1 and 2 combined, which ranked the 1st place on the task. Our results showed that an SPBA trained on an external NER dataset achieved reasonable performance on a partial matching evaluation metric. The CRF-based recognizer trained on GPRO mentions achieves high performance on the GPRO task. However, the performance of GPRO recognition is further improved by using SPBA patterns as features.

## Related work

In this section, we briefly review state-of-the-art GPRO recognition systems and SPBA-related work.

### Gene and protein related object

The GPRO recognition task was first included in BioCreative V [4], where the top-performing system was developed by [5]. They combined the results of five recognizers by majority voting method. All recognizers were CRF-based but used different combinations of GPRO mention types and features, which were adapted from GNormPlus features [6]. In addition, [5] employed some heuristic post-processing steps like enforcing tag consistency and full-abbreviation. Also, a maximum-entropy (ME)-based filter was developed to remove false positive predictions. They achieved an F-score of 81.37% in the BioCreative V GPRO task.

In the BioCreative V.5 GPRO task, [7] used a BiLSTM (Bidirectional Long Short-Term Memory) model to identify gene and protein related objects. The BiLSTM architecture was the same as that used by [8]. The word embedding consisted of character-level and token-level representations, and bidirectional LSTM was used to generate character-level embedding from the characters of a word. The input embedding of characters was randomly initialized. Character-level representation could capture the morphology of words like prefixes and suffixes. Then a word embedding layer was used as the input for the next bidirectional LSTM layer. Using bidirectional LSTM layers could capture the context information of the current token. Following the bidirectional LSTM layer was a CRF layer which was able to learn the label transition states of GPRO labels. Their system achieved F-scores of 76.34% and 75.91% on the GPRO Type 1 and GPRO Type 1 + 2 evaluation metrics, respectively. Luo et al.’s [9] approach was basically the same as Liu et al. [7]; however, [9] achieved a higher F-score of 79.19% on the GPRO Type 1 evaluation metric compared to Liu et al. [7] 76.34%. Luo et al.’s [9] system also achieved an F-score of 72.28% on the GPRO Type 1 + 2 evaluation metric. The lower performance on the GPRO Type 1 + 2 metric mainly resulted from the failure of their system to identify many Type 2 GPRO mentions (false negative).

### Statistical principle-based approach

SPBA is a straightforward, easy-to-interpret framework for resolving natural language processing (NLP) problems such as question answering or topic classification. SPBA consists of three main parts: semantic map/ontology, principle generation, and principle matching. SPBA was first used to solve tasks in general domains such as sentiment classification of Chinese news [10] and answering restaurant-related questions [11]. SPBA has been adapted for biomedical tasks, including miRNA recognition [12], miRNA-target interaction extraction [13], and gene-metastasis relation extraction [14].

## Methods

**Fig. 1:**
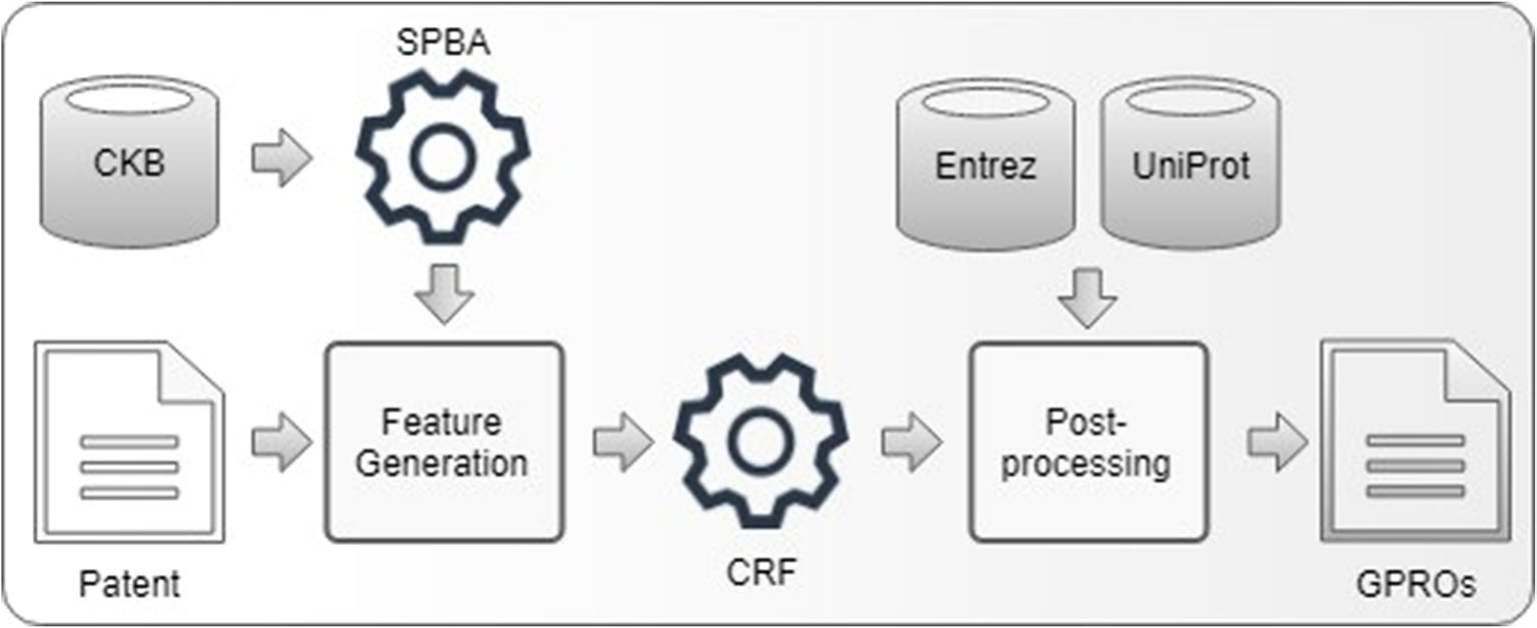
The workflow of SPBA-CRF system

In this section, we describe SPBA-CRF in more details. SPBA-CRF consists of three stages: SPBA,^1^ CRF, and post-processing. Figure 1 shows the flowchart of the whole system. First, SPBA is trained on a revised version of the JNLPBA dataset, and then employed to identify gene mentions. Following SPBA, we train a CRF-based GPRO recognizer on the GPRO training set. The predictions of SPBA are used as one of the features for the GPRO recognizer. Finally, our post-processing module refines the boundaries and the type for each GPRO mention if necessary. All states are detailed in the following subsections.

### Statistical principle-based approach

**Fig. 2:**
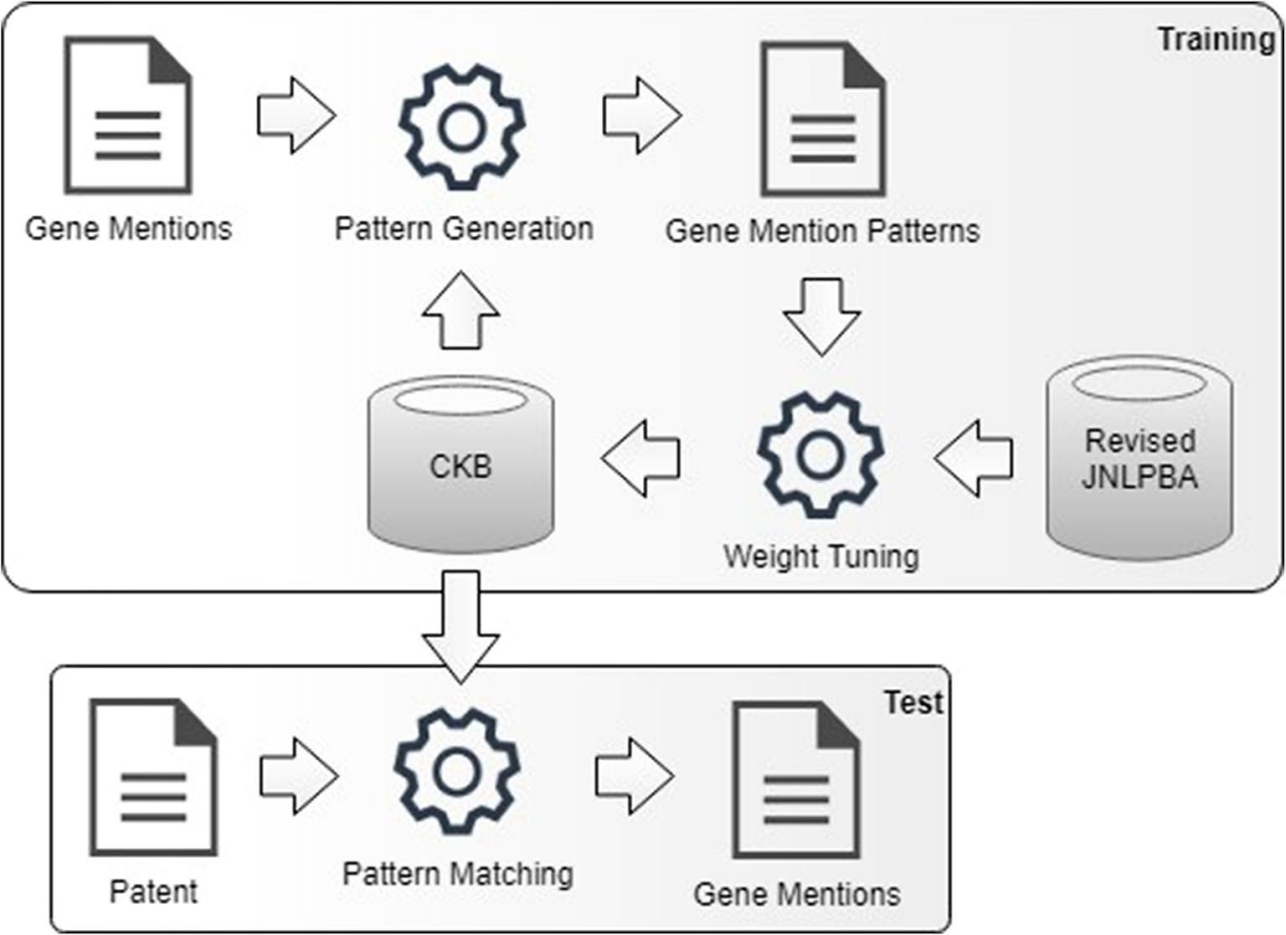
The flowchart of SPBA system

Figure 2 illustrates the flowchart of the training and test procedures of SPBA. SPBA employs automatically generated patterns with learned weights to identify NEs. The training stage of SPBA contains two steps: pattern generation and weight tuning.

In SPBA, patterns describe the formation of an NE type and are used to match mentions of that NE type. An SPBA pattern is composed of words and entities defined in a concept knowledge base (CKB). For gene mention type, our domain experts constructed a CKB containing vocabularies collected from several public resources (as shown in Table 2). To prepare the data for generating patterns of the gene mention type, we used the CKB to label all gene mentions in the revised JNLPBA training set.

**Table 2:** Concept Knowledge Base (CKB)

Class	Name	Description	Examples
Attribute	BiologicalProcess	GeneOntology	*“apoptosis”*, *“inhibitory”*
	Chemical	ChEBI	*“glucose”*, *“sodium”*
	Disease	MeSH term	*“leukemia”*, *“tumor”*
	Morphology	Keyword	*“mononuclear”*, *“fibroblastic”*
	OrganTissue	SWISS-PROT	*“kidney”*, *“mesenchymal”*
	Taxonomy	NCBI Taoxonomy	*“feline”*, *“murine”*
	Structure	ExPAsy	*“motif”*, *“zinc finger”*
NEKeyword	CellKeyword	Regular expression	*“cell”*, *“lymphocyte”*
	CellLineKeyword	Regular expression	*“cell line”*, *“clone”*
	DNAKeyword	Regular expression	*“DNA”*, *“promoter”*
	ProteinKeyword	Regular expression	*“protein”*, *“factor”*
	RNAKeyword	Regular expression	*“mRNA”*, *“transcript”*
Symbol	CellLineSymbol	CLDB	*“A549”*, *“BFTC905”*
	CellTypeSymbol	ExPAsy	*“PBMC”*, *“HUVEC”*
	ChromosomeSymbol	Regular expression	*“11p15”*, *“14q32.1”*
	GeneSymbol	Entrez and PubTator	*“TNF alpha”*, *“VEGF”*
Others	Conjunction	GENIATagger	*“and”*, *“or”*
	Preposition	GENIATagger	*“in”*, *“of”*
	Specifier	Regular expression	*“alpha”*, *“I”*

In the weight tuning step, we use the CKB to label sentences in the revised JNLPBA training set. Then, for each pattern *p*, we match *p* with the labeled sentences. A logistic regression model [15] to tune the weights for different matching features. The vector of weights **W** resulting in the least log loss value is selected. In the test stage, unseen sentences are labeled by the CKB. Then, the generated SPBA patterns with the tunned weights are used to identify NEs.

*Concept Knowledge Base* An NE is composed of one or more words. Some of these words could be generalized to concepts. For example, *“nitric oxide”* could be generalized to the *“Chemical”* concept. If we express a NE as a set of sequences of concepts (called pattern), these patterns are likely to match unseen instances of that NE type. We construct a CKB to collect element entities forming a type of NEs by collecting the concept set from publicly available biological databases shown in Table 2. In addition to using the official gene synonyms of Entrez, we also used the manually-curated NEs of PubTator [16].

*Pattern Generation* To generate patterns, we first employ prefix-tree matching to label all NEs in the training set by using the CKB. Then, unlabeled words are removed, and the remaining label sequence is called a pattern. Since an NE may be labeled in more than one way, generating more than one patterns, we only keep the pattern with the highest labeled ratio (the number of labeled words/the number of words). Table 3 illustrates the examples of NEs and patterns.

**Table 3:** The examples of generated patterns

CKB-labeled gene mention	Generated pattern
*“* ***38-kD***_***Unit***_ ***murine***_***Taxonomy***_ ***MAP***_***GeneSymbol***_ ***kinase***_***Enzyme***_ *”*	[Unit][Taxonomy][GeneSymbol][Enzyme]
*“* ***BanHI***_*GeneSymbol*_ ***A***_***Specifier***_ *rightward* ***transcripts***_***RNAKeyword***_ *”*	[GeneSymbol][Specifier][RNAKeyword]
*“* ***human***_***Taxonomy***_ ***M-CSF***_***GeneSymbol***_ ***promoter***_***ProteinKeyword***_ *”*	[Taxonomy][GeneSymbol][ProteinKeyword]
*“* ***nitric oxide***_***Chemical***_ ***synthase***_***Enzyme***_ ***mRNA***_***RNAKeyword***_ *”*	[Chemical][Enzyme][RNAKeyword]
*“****phosphatase***_***Enzyme***_ ***2A***_***Specifier***_-*sensitive* ***genes***_***DNAKeyword***_*”*	[Enzyme][Specifier][DNAKeyword]
*“* ***transcription factor***_***ProteinKeyword***_ ***NF*** **-** ***kappaB***_***GeneSymbol***_ *”*	[ProteinKeyword][GeneSymbol]

**Fig. 3:**
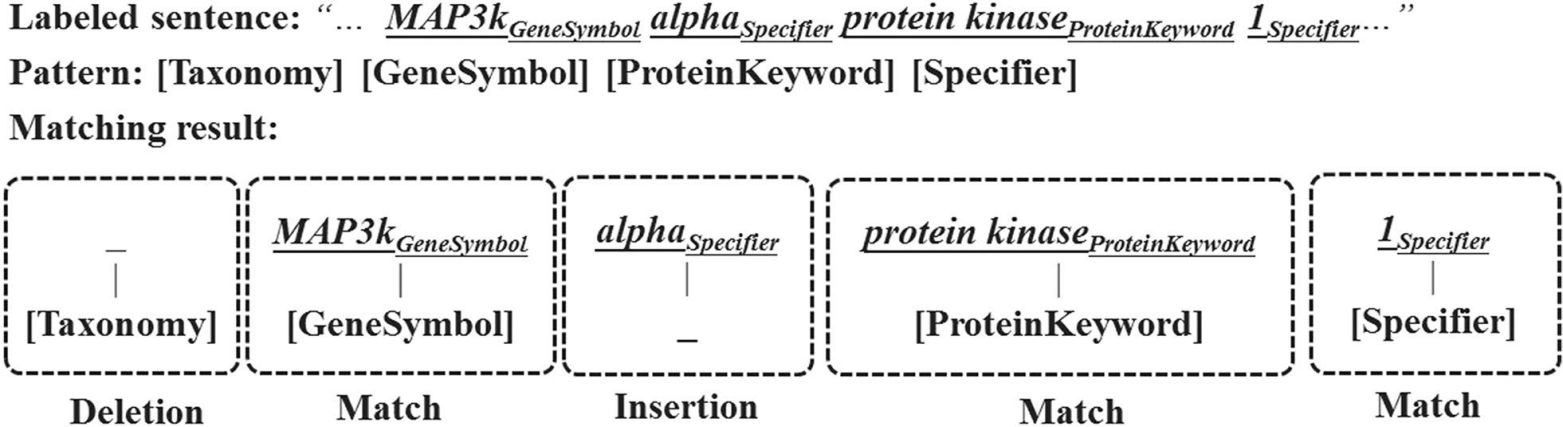
An example of successful pattern matching

*Pattern Matching* After pattern generation, the patterns will be used to recognize candidate NEs in sentenses labeled by a CKB. Our pattern matching method is similar to regular expression matching. A successful matching allows insertion of words and deletion of concepts. The Fig. 3 presents an example of successful pattern matching. For each pattern, the scoring of matching is based on its features and its tuned vector of weights. The features used for scoring a matching result are illustrated in Table 4.

**Table 4:** The features for scoring a pattern matching result

Feature set	Description
Alignment feature set	Insertion/deletion/matching words/concepts
Context feature set	Surrounding words and POS tags
Singleton feature set	Morphology

### Conditional random fields-based recognizer

**Fig. 4:**
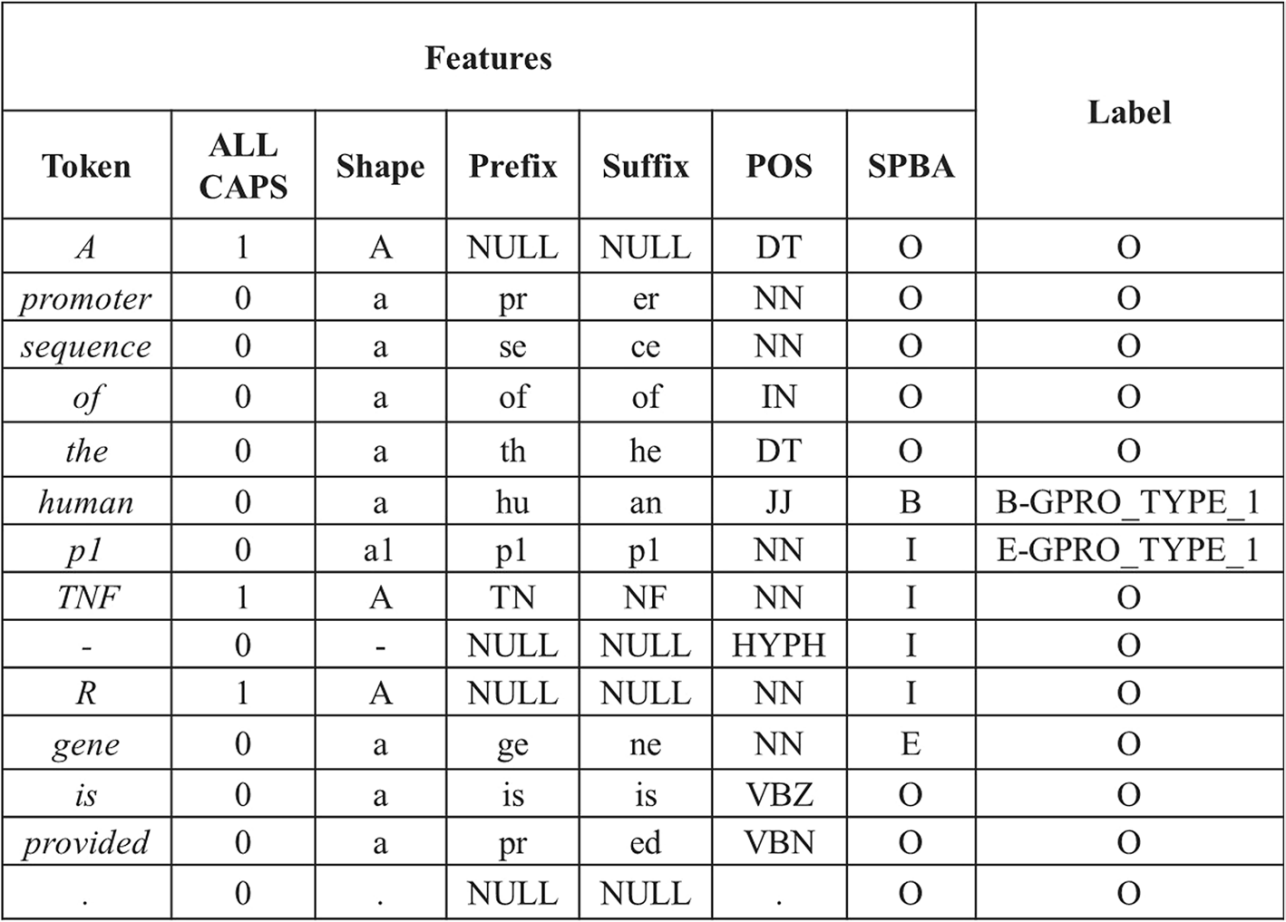
An example of CRF features

There are two differences between SPBA’s annotations and GPRO’s. The first difference exists in their NE classifications. SPBA contains five NE types: cell line, cell type, DNA, protein and RNA. The GPRO task has two NE types: Type 1 and 2. The second difference exists in their NE spans. SPBA prefers to annotate longer phrase/chunk as NEs, however GPRO task prefers to use the phrase/chunk which could exactly match the database’s official name. Thus, we find that GPRO mentions were usually substrings of SPBA’s NEs. To identify GPRO mentions, we employ our previous chemical name recognizer, NERChem [17], which bases on the CRF model. Firstly, we employ the GENIATagger [18] to segment every sentence into a sequence of tokens. Then, we run a sub-tokenization module used in our previous work [17] to further segment tokens into sub-tokens. We use the SOBIE tag-scheme which has nine labels including B-GPRO_TYPE_1, I-GPRO_TYPE_1, E-GPRO_TYPE_1, S-GPRO_TYPE_1, B-GPRO_TYPE_2, I-GPRO_TYPE_2, E-GPRO_TYPE_2, and S-GPRO_TYPE_2, and O. The characters B, I, E, S and O represent the beginning of a mention, inner of a mention, end of a mention, singleton, and otherwise, respectively. We use features including word, POS, affix, orthographic, word shape and chunk features. For word features, we normalize every single digit letter. We also use the labeling results of SPBA as features. Figure 4 shows an example of our features.

### Post-processing

The post-processing stage is used to refine GPRO mentions’ spans and type. It includes four steps: ID mapping, enforcing tag consistency, applying GPRO annotation standard, and FP filtering. Below we will introduce each step.

*ID Mapping* We adjust the mention type of a GPRO mention according to whether the GPRO mention could be assigned to unique database ID through ID mapping process. We first build a mapping table to map NE to its database ID(s). The table is constructed by using protein records of UniProt database. To allow more mentions mapped to their IDs, we use heuristic rules [19], like converting to lower cases, removing the symbols, removing the named entity suffix *“s”*. If two or more matching IDs are found, we use the Entrez homolog dictionary to normalize homolog IDs to human IDs. If a mention has exactly one ID, it is labeled as Type 1. Otherwise, it is labeled as Type 2.

*Enforcing Tag Consistency* To ensure the consistency of tag, we first collect recognized GPRO mentions as well as pairs of GPRO mentions and their abbreviations in a given document. Then, we use the maximum matching algorithm to find missing GPRO mentions.

### Applying GPRO annotation standard

Full-Abbreviation rule: If the keyword *“receptor”* follows a pair of a full GPRO name and its abbreviation, we will merge the pair and extend the right of the span to the end of the keyword. For example, *“****epidermal growth factor***_***GPRO***_ *(****EGF***_***GPRO***_*) receptor”* will be *“****epidermal growth factor (EGF) receptor***_***GPRO***_*”.*

Short name (1–2 token(s)) rules: Since there are many short GPRO mentions (approximately 85% in the GPRO training set), we design two rules to adjust the spans of recognized GPRO mentions.

- If a recognized two-token GPRO mention ends with *“protein”* and the character length of the GPRO abbreviation is larger or equal to 3, we will discard the *“protein”*. For example, *“****p53 protein***_***GPRO***_*”* will be *“****p53***_***GPRO***_ ***protein****”.*
- If a single-word GPRO mention is followed by *“protein”* and the GPRO mention is shorter than 3 characters, we will expand the right span to the end of *“protein”*. For example, *“****AR***_***GPRO***_ *protein”* will be *“****AR protein***_***GPRO***_*”.*

*FP Filtering* The CTD [20] chemical dictionary and the DrugBank [21] drug dictionary are used as the blacklist of GPRO mentions.

## Experiment results

Our experiments are conducted on the Biocreative V.5 GPRO dataset. The evaluation script of BeClam [22] is used, and we find that the evaluation script of BeClam is combining GPRO Type 1 and 2 instead of only GPRO Type 1 which is used in Biocreative V GPRO task [4].

We use four different evaluation metrics, (1) strict F1-measure of Type 1, which is the same as Biocreative V GPRO task, (2) strict F1-measure of combining Type 1 and 2, which is the same as BeClam, (3) relaxed F1-measure of Type 1, which allows the spans of predicted GPRO mentions and gold GPRO mentions to be partially matched, (4) relaxed F1-measure of combining Type 1 and 2. We present the performance of three experiments. The first experiment examines the effect of using SPBA only. In the next experiment, we observe the influence of adding CRF. Lastly, the performances of our submissions are reported.

### Dataset

The Biocreative V.5 GPRO dataset is used to evaluate our approach. It contains patents from 2005 to 2014 that have been assigned either the A61P1 or A61K31 2IPC (International Patent Classification) codes, meaning the patents are relevant to medical chemistry and mention synthetic organics.

The Biocreative V.5 GPRO dataset contains the training and test set. The training set contained 21,000 patent abstracts, and the test set contains 7000. Since they do not provide additional development set. We use two-fold cross-validation to evaluate our system in our system development stage.

The task uses the same evaluation metric with the Biocreative V GPRO task. Furthermore, they also report the performances of combining Type 1 and 2 in official result.

### Experiment 1

In experiment 1, we evaluate the performances of SPBA in terms of both the strict and relaxed evaluation metrics. Since SPBA can not classify the GPRO type, we only report the performances of combining Type 1 and 2. The performances are shown in Table 5.

**Table 5:** The performances of SPBA on strict and relaxed evaluation metrics

Evaluation	Prec. (%)	Rec. (%)	F-score (%)
Strict	58.4	63.8	61.0
Relaxed	79.0	88.5	83.5

Although SPBA achieves only an F-score of 61.0% in terms of the strict metric, it achieves an F-score of 83.5% in terms of the relaxed metric, showing that the SPBA method achieves reasonable performance if the purpose of NER does not require strict boundary identification. Notice that although the span definition of NE in the GPRO and revised JNLPBA datasets are very different, SPBA achieves a very high recall in partial matching evaluation metric.

### Experiment 2

In experiment 2, we evaluate the effect of integrating SPAB and CRF. The performances are shown in Tables 6 and 7. Table 6 shows the performances of our two configurations. The first configuration (CRF) is the CRF model with baseline features. The second configuration (SPBA-CRF) is the CRF model with baseline features plus SPBA features. The SPBA-CRF recognizer outperforms the CRF recognizer by F-scores of 3.6% and 3.1% in Type 1 and the combining one respectively. The improvement is brought majorly from the improved recall, indicating that SPBA can help CRF to identify more GPRO mentions without losing precision.

**Table 6:** The performances of CRF and SPBA-CRF on the strict evaluation metric

Configuration	Type 1			Combining Type 1 and 2		
	Prec. (%)	Rec. (%)	F-score (%)	Prec. (%)	Rec. (%)	F-score (%)
CRF	71.1	72.9	72.0	76.7	76.3	76.5
SPBA-CRF	71.8	77.5	75.6	78.5	80.8	79.6

**Table 7:** The performances of SPBA-CRF on the relaxed evaluation metric

Configuration	Type 1			Combining type 1 and 2		
	Prec. (%)	Rec. (%)	F-score (%)	Prec. (%)	Rec. (%)	F-score (%)
SPBA-CRF	79.8	86.2	82.9	89.0	92.0	90.5

In the relaxed evaluation metric, our SPBA-CRF achieves an F-score of 82.9% on the Type 1 as shown in Table 7. If we combine Type 1 and 2, SPBA-CRF can achieve an F-score of 90.5%.

### Experiment 3

Table 8 shows the performance of our submissions to the BioCreative V GPRO task, both of them are SPBA-CRF. The config. 1 uses the ID mapping of the post processing and config. 2 does not. In config. 1, the type 2 NEs are removed from our submission. It seems that the ID mapping increases the precision but decreases the recall. Therefore, the config. 2 slightly outperforms the config. 1. Our best configuration achieves an F-score of 73.73% on Type 1, and an F-score of 78.66% in terms of the combining metric of Type 1 and 2.

**Table 8:** The performances of our submissions in the test set

Configuration	Type 1			Combining type 1 and 2		
	Prec. (%)	Rec. (%)	F-score (%)	Prec. (%)	Rec. (%)	F-score (%)
1. SPBA-CRF	68.69	78.24	73.15	81.44	74.67	77.91
2. 1 without ID mapping	66.53	82.68	73.73	78.63	78.70	78.66

## Discussion

### The improvement of adding SPBA as feature

Table 6 shows that adding SPBA improves a recall of 4.6% and 4.5% in Type 1 and Type 1 + 2 respectively. According to our observation on these cases, approximately 54% GPRO mentions are missed by the CRF-based recognizer can be exactly identified by SPBA. Furthermore, approximately 28% GPRO mentions missed by the CRF-based recognizer can be partially identified by SPBA. Therefore adding SPBA as features can help CRF-based recognizer to identify more GPRO mentions.

### The lower precision of SPBA-CRF

Table 8 shows that SPBA-CRF has lower precision in the GPRO Type 1 evaluation metric. Since the gold annotations of test set are not available, we conduct an error analysis on the training set. The lower precision mainly comes from two reasons. First, SPBA-CRF often predicts longer spans of GPRO mention than the spans of gold GPRO mention. There are approximately 13% false positive cases come from inconsistent spans of predicted and gold GPRO mentions. Another reason is that SPBA-CRF sometimes fails to distinguish Type 1 and Type 2. There are approximately 30% false positive cases come from that SPBA-CRF classified Type 2 GPRO mentions into Type 1 GPRO mentions.

## Conclusion

In this paper, we have described the construction of an SPBA-CRF-based system that can automatically recognize GPRO mentions in chemical patents. Our system uses SPBA NE predictions as features for a CRF-based GPRO recognizer, and uses the post-processing methods to adjust GPRO mention spans and mention types. Experimental results show that SPBA achieves reasonable performance in partial matching evaluation. Furthermore, adding the SPBA NE predictions as CRF features boosts the F-score from 76.5% (baseline features) to 79.6%. This demonstrates that SPBA helps the CRF-based recognizer to identify more GPRO mentions without decreasing precision. We evaluate our system on the BioCreative V.5 GPRO task, and SPBA-CRF achieves an F-score of 73.73% on GPRO Type 1, which is ranked the 4th place overall, and an F-score of 78.66% on GPRO Type 1 + 2, giving our system the top-ranked position.

## Footnotes

1 SPBA, Concept Knowledge Base and revised JNLPBA dataset are avaliable in the website http://iasl-btm.iis.sinica.edu.tw/bner.
